# He–Ne laser irradiation enhances curcuminoid content in *Curcuma longa*: a green strategy for functional antioxidants and antimicrobial ingredients

**DOI:** 10.3389/fnut.2026.1758942

**Published:** 2026-02-18

**Authors:** Munirah F. Aldayel

**Affiliations:** Biological Sciences Department, College of Science, King Faisal University, Al-Ahsa, Saudi Arabia

**Keywords:** antioxidant activity, *Curcuma longa*, curcuminoids, functional ingredients, He–Ne laser, laser irradiation, natural antimicrobials, sustainable processing

## Abstract

**Introduction:**

*Curcuma longa* rhizomes are rich in curcuminoids with strong antioxidant and antimicrobial activities. Sustainable post-harvest strategies are needed to enhance these bioactive metabolites for functional food applications.

**Methods:**

Fresh rhizomes were irradiated with a He–Ne laser for 5, 10, or 15 min. Ethanol extracts were analyzed by High Performance Liquid Chromatography analysis (HPLC) to quantify curcuminoids. Antioxidant activity was evaluated using 2,2-diphenyl-1-picrylhydrazyl (DPPH) assays, and antibacterial activity tested against *Escherichia coli* (*E. coli*), *Staphylococcus aureus* (*S. aureus*), and *Streptococcus pyogenes* (*S. pyogenes*).

**Results:**

Laser treatment increased curcuminoid content in a time-dependent manner, with 15-min exposure yielding the highest bisdemethoxycurcumin enrichment. Antioxidant assays showed strong scavenging potential, with IC50 values of 0.71 (10-min) and 0.69 mg mL^−1^ (15-min). Extracts exhibited enhanced antibacterial activity, with 15-min exposure strongest against *E. coli* and *S. aureus*, while 10-min treatment yielded higher inhibition of *S. pyogenes*.

**Discussion:**

He–Ne laser irradiation is an effective green technique to improve the antioxidant and antimicrobial properties of *C. longa* extracts, supporting their use as natural functional ingredients.

## Highlights

- He–Ne laser irradiation enhances the bioactive metabolite profile of *Curcuma longa* (*C. longa*) in a clean and non-thermal manner
- Laser-treated extracts show stronger antibacterial activity against *E. coli*, *S. aureus*, *S. pyogenes*, and MRSA
- Curcuminoid and phenolic enrichment results in higher antioxidant capacity
- Extracts remain non-cytotoxic at effective MIC levels, supporting safe application
- The approach provides a sustainable method to develop potent natural antimicrobial and antioxidant ingredients for functional foods and nutraceuticals

## 1 Introduction

Globally known as a culinary spice and medicinal plant, turmeric (*Curcuma longa L*.) is rich in curcuminoids, mainly curcumin, demethoxycurcumin, and bisdemethoxycurcumin, which have antibacterial, anti-inflammatory, and antioxidant properties (1, 2). Nevertheless, the natural curcumin output from dried rhizomes is still limited (usually less than 3% w/w), which restricts its effective use in industrial applications and value-added functional ingredients (3). A major obstacle to the scalable and economical manufacturing of natural colorants, preservatives, and nutraceuticals is the extractable bioactive content bottleneck.

Clean-label preservation techniques are becoming more and more necessary in the food sector to replace synthetic antioxidants and antimicrobials like BHA, BHT, and nitrites, whose use is decreasing as a result of consumer concerns and regulatory pressures (4, 5). The need for natural antimicrobial compounds with broad activity and low toxicity has been highlighted by the increasing prevalence of multidrug-resistant (MDR) organisms in food systems (6, 7), such as *Staphylococcus aureus*, *methicillin-resistant S. aureus* (*MRSA*), *E. coli*, and *Klebsiella pneumoniae* (8, 9). Although curcuminoids are interesting possibilities, their industrial viability is limited by their low natural abundance and vulnerability to thermal degradation during processing (10–12).

Non-thermal elicitation techniques have drawn interest as a way to get around these limitations since they can promote the accumulation of secondary metabolites without requiring a lot of energy or chemical inputs (13). Helium–neon (He–Ne) laser irradiation is one of the new methods that has been shown to alter metabolic pathways in post-harvest tissues, possibly improving curcuminoid biosynthesis while maintaining structural integrity (14–16). Turmeric may become a more powerful ingredient in functional foods, nutraceuticals, and natural antibacterial formulations thanks to such green processing approaches, which also correspond with worldwide aspirations for sustainable (17) residue-free augmentation of bioactive chemicals (18).

Growing interest in “green processing” and “clean-label” technologies (19) to extract and valorize plant-derived bioactive compounds has been observed in recent years. These technologies aim to minimize energy consumption, solvent use, and environmental impact while maintaining or improving bioactivity and suitability as functional ingredients (20, 21).

These cutting-edge technologies include moderate or non-thermal processing techniques as light irradiation, pulsed electric fields, supercritical fluid extraction, and ultrasound-assisted extraction (22–24). Of these, light-based post-harvest treatments—specifically, UV, LED, or laser irradiation—have lately come to light as a viable, environmentally friendly way to promote the buildup of bioactive metabolites in foods or by-products derived from plants without using harsh chemicals or high temperatures (25–27). For example, it has been demonstrated that post-harvest light or UV treatments increase antioxidant molecules and enhance the nutritional and functional quality of fruits and vegetables while they are being stored (28–30). More generally, the food industry is paying more attention to laser technology because of its potential for processing, microbial inactivation, surface sterilization, packaging, and improving the extractability of bioactive compounds. It provides a contactless, accurate, and non-thermal tool for sustainable food processing (31). In light of these developments, we hypothesized that post-harvest irradiation of turmeric rhizomes with a low-power laser (He–Ne) might be used as a “green processing” technique to increase curcuminoid levels and thereby improve the functional antioxidant and antimicrobial potential of turmeric extracts, opening the door for their use as natural preservatives or clean-label functional ingredients in food or nutraceutical applications (32, 33). Laser pulse therapy has the potential to greatly impact and improve the antibacterial effectiveness of turmeric extracts (34). This integrated strategy makes use of laser energy’s modifying effects as well as turmeric’s natural antibacterial qualities (35, 36). However, to the best of our knowledge, no prior research has examined the influence of He–Ne laser irradiation on curcuminoid accumulation in turmeric rhizomes or the ensuing effects on the extracts’ antioxidant and antibacterial activity. Instead of focusing on secondary metabolite increase for food-ingredient valorization, previous laser-based research have mainly examined seed germination, plant growth, or yield (16, 33, 37–39).

In order to ascertain if post-harvest He–Ne laser irradiation can enhance important curcuminoids in *C. longa* rhizomes, this study also examines the associated impacts on antioxidant capacity and antibacterial activity against bacteria that are relevant to food and MDR bacteria. This effort attempts to connect phytochemical enhancement with real-world food business needs for safer, natural, and sustainable preservation systems by connecting metabolite enrichment with functional bioactivity outcomes.

The current work sought to determine if post-harvest He–Ne laser irradiation may improve the bioactive metabolite content and associated activities of *Curcuma longa* extracts, hence boosting their functional value. In particular, the goals were to: Calculate the changes in the main curcuminoids (curcumin, demethoxycurcumin, and bisdemethoxycurcumin) after exposure to a He–Ne laser at various irradiation times. Using DPPH radical scavenging experiments, determine whether laser treatment increases the antioxidant capacity of ethanolic turmeric extracts. Analyze the antibacterial activity of laser-treated extracts against food-relevant and multidrug-resistant (MDR) bacterial strains, such as *MRSA*, *E. coli*, *S. aureus*, and *S. pyogenes*. To confirm the bacteriostatic or bactericidal properties of enriched extracts, calculate the minimal inhibitory and bactericidal concentrations (MIC/MBC). Use scanning electron microscopy to investigate the impact of cells on bacterial morphology in order to establish a correlation between biochemical stimulation and structural damage. Examine the potential of laser-enhanced turmeric extracts as clean-label functional additives for sustainable food applications and natural preservation.

## 2 Materials and methods

### 2.1 Plant material and post-harvest He–Ne laser treatment

Fresh rhizomes of *Curcuma longa* (*C. longa*) were sourced from SEKEM (Heliopolis, Cairo, Egypt). Air-dried rhizomes were exposed to helium–neon (He–Ne) laser irradiation (*λ* = 632.8 nm) at a power density of 5.5 mW mm^−2^, with a fixed source–sample distance of 40 mm. Three exposure durations were applied: 5, 10, and 15 min.

### 2.2 Greenhouse cultivation

Rhizomes from each treatment were grown in the Agriculture & Veterinary Research Center (King Faisal University, Saudi Arabia). Plants were maintained under controlled environmental conditions (32–36 °C, 47–56% RH, 14 h photoperiod). After 8 months, rhizomes were harvested, air-dried, and ground for extraction.

### 2.3 Preparation of ethanolic extracts

Dried rhizome powder (5 g) was extracted in 50 mL ethanol by maceration for 4 days at room temperature with periodic shaking. Extracts were filtered (0.22 μm) and concentrated under reduced pressure at 40 °C.

### 2.4 High performance liquid chromatography analysis (HPLC) quantification of curcuminoids

Dried ethanolic residues were reconstituted in 5.0 mL of mobile phase (acetonitrile: 0.1% trifluoroacetic acid in water, 1:1, v/v; TFA present in both solvents). Reconstituted solutions were centrifuged (3,000×*g*, 5 min) and filtered through 0.22 μm syringe filters prior to injection. Chromatographical analysis were completed on a Waters 2,690 Alliance HPLC system, equipped with a photodiode array detector (Waters 996). Separation was achieved on a C18 Inertsil column (4.6 × 250 mm, 5 μm) with controlled temperature (25 °C) by a mobile phase, delivered isocratically at 1.0–1.5 mL per minute. The sample volume (10 μL) and/or standard was inserted, and detection was performed at 420 nm with full spectral acquisition (200–600 nm) for peak confirmation. Standards of curcumin (≥99.5%), demethoxycurcumin (≥98%) and bisdemethoxycurcumin (≥98%) were obtained from Sigma-Aldrich (St. Louis, United States). Stock solution (1 mg mL^−1^) was prepared in methanol and diluted to produce working concentrations (10, 20, 30, 40, 50 and 60 μg mL^−1^) whereas, standard solutions filtered through syringe filters (0.22 μm) prior to injection were used. Calibration curves were constructed from peak areas and used for quantification; linearity (*R*^2^), limits of detection and quantification, and method precision were measured during method validation. The mobile phase was filtered (0.45 μm) and degassed under vacuum before use. System suitability (retention time repeatability, theoretical plates, tailing factor and resolution) and periodic quality control injections were performed throughout analytical runs (19).

### 2.5 Characterization of laser irradiation

#### 2.5.1 UV–visible spectroscopy

The color change was verified with UV–visible spectroscopy (Shimadzu 8,400, Kyoto, Japan) between 300 and 700 nm wavelength range was utilized for the analysis (20).

#### 2.5.2 X-ray diffraction analysis

The EDX technique was used to determine the elemental makeup of the synthesized silver nanoparticles. The test was conducted in an X-ray diffractometer (Unisantis XMD-300, Swiss, Basel, Switzerland) (20).

#### 2.5.3 Fourier transform infrared spectroscopy (FTIR)

This analysis was used to characterize the functional groups involved in the formation and stabilization of the biosynthesized He–Ne laser irradiation (40). The analysis was performed using an FTIR-8400 spectrometer (Shimadzu, Kyoto, Japan), with measurements.

recorded in the range of 500–4,500 cm^−1^. This allowed identification of key chemical bonds and functional groups associated with the He–Ne laser irradiation (21).

#### 2.5.4 Energy dispersive X-ray spectroscopy (EDX)

The EDX technique was used to determine the elemental makeup of the synthesized He–Ne laser irradiation. The collected dried He–Ne laser irradiation synthesized from *C.longa* (previously described) were analyzed using a scanning electron microscope integrated with an EDX (Tescan, Brno, Czech Republic) (20).

#### 2.5.5 Zeta potential

Zeta potential and particle size distribution measurements were conducted using a spectrophotometer from Malvern Analytical, Enigma Business Park, UK. using Zetasizer 7.01 software was used to observe the surface charge of the He–Ne laser irradiation (22).

### 2.6 Bacterial strains and culture conditions

Clinical isolates (*E. coli*, *S. aureus*, *MRSA*, *S. pyogenes*) were obtained from King Faisal University. Overnight cultures in nutrient broth (NB) were adjusted to OD600 = 0.3 (~1 × 10^8^ CFU·mL^−1^ ≈ 0.5 McFarland) (2).

### 2.7 Disc diffusion assay

Sterile 6-mm discs were loaded with extracts to achieve 5 mg·L^−1^ curcuminoid equivalents per disc. Imipenem (10 μg/disc) served as positive control; 1:1 DMSO: water served as negative control. Plates were incubated at 37 °C for 18–24 h (2).

### 2.8 Minimum inhibitory concentration (MIC) and minimum bactericidal concentration (MBC)

Serial dilutions of extracts (0.125–4 mg·mL^−1^) were prepared in NB. MIC was defined as the lowest concentration yielding no visible growth after incubation at 37 °C. MBC was confirmed by plating MIC-negative wells and observing colony absence after 24 h at 37 °C (2).

### 2.9 Scanning Electron microscopy

Cells treated with extract (MIC level) for 4 h were fixed in 2.5% glutaraldehyde, dehydrated, dried, sputter-coated, and visualized at 20,000 × magnification (23).

### 2.10 Antioxidant activity: DPPH assay

Extracts were tested at 0.2–1.0 mg·mL^−1^ against DPPH (517 nm), with gallic acid as standard. IC₅₀ values were derived from regression analysis (2).

### 2.11 Protein denaturation anti-inflammatory assay

Egg albumin was incubated with extract (up to 500 μg·mL^−1^) in phosphate buffer and heated to 70 °C. Absorbance at 660 nm was used to calculate inhibition (41).

### 2.12 Cytotoxicity assay

MCF-7 cells were treated with extract concentrations up to 200 μg·mL^−1^ for 48 h, followed by Mean Transit Time (MTT) assay (550 nm) (2).

### 2.13 Statistical analysis

Data were analyzed using one-way ANOVA with Tukey’s Honestly Significant Difference (HSD) (*p* ≤ 0.05). Results reported as mean ± SD of biological triplicates.

## 3 Results

### 3.1 High performance liquid chromatography analysis (HPLC)

After 3 months of cultivation, all plants derived from rhizomes exposed to 5 min He–Ne laser irradiation consistently exhibited early rhizome yellowing followed by complete plant death across two independent planting seasons. As a result, no growth or HPLC data could be obtained for this treatment. This reproducible response suggests that short-duration laser exposure may induce acute photo stress or transient oxidative imbalance sufficient to impair tissue viability as has been reported for brief laser treatments in other plant systems (42), without triggering adaptive metabolic responses observed at longer irradiation durations. He–Ne laser irradiation significantly altered curcuminoid composition in *C. longa* rhizomes (Table 1). Bisdemethoxycurcumin increased by 42–43% following 10–15 min exposure compared with control (*p* ≤ 0.05). Demethoxycurcumin decreased after 10 min irradiation and partially recovered at 15 min but remained lower than control values (*p* ≤ 0.05). Curcumin levels were reduced at 10 min but maintained at levels statistically comparable to control after 15 min irradiation (p ≤ 0.05). Variability across treatments exhibited structure-dependent responses to irradiation duration.

**Table 1:** Curcuminoid concentrations (μg mL^−1^; mean ± SD, *n* = 3) in *C. longa* extracts following He–Ne laser treatment.

Treatment	Bisdemethoxycurcumin	Demethoxycurcumin	Curcumin
Control	70.76 ± 2.1^b^	80.40 ± 2.5^a^	327.25 ± 5.3^a^
10 min Laser	100.50 ± 3.2^a^	60.78 ± 1.9^c^	220.85 ± 4.1^b^
15 min Laser	101.19 ± 3.1^a^	70.68 ± 2.0^b^	380.80 ± 6.2^a^

^*^Different superscript letters within a column indicate significant differences (*p* ≤ 0.05, Fisher’s LSD).

Curcuminoid responses to laser irradiation were structure-dependent and non-linear. Bisdemethoxycurcumin showed a consistent and significant increase following 10–15 min irradiation, whereas curcumin and demethoxycurcumin exhibited differential responses, likely reflecting competing biosynthetic and photo-degradation processes.

### 3.2 He–Ne laser irradiation enhances curcuminoid content in *Curcuma longa*: physicochemical characterization (FTIR, XRD, SEM–EDX, zeta potential) and functional bioactivity

#### 3.2.1 UV–visible spectral analysis

**Figure 1:**
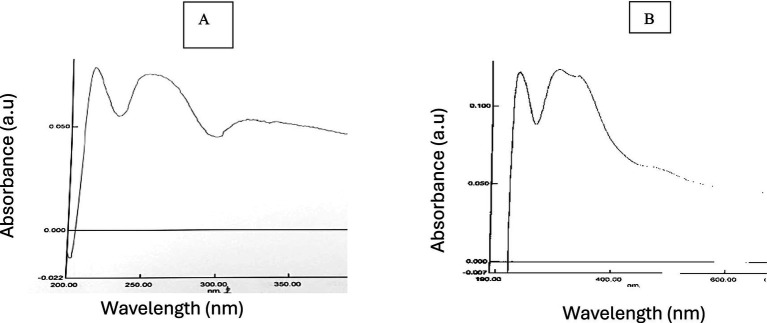
UV–Vis absorption spectrum of **(A)** 10 min laser irradiation of *C. longa*, **(B)** 15 min laser irradiation of *C. longa.*

The formation of *C. longa* was determined by UV–visible spectroscopy, as shown in (Figure 1). The results reveal a higher absorbance at 335 nm in 10 min laser irradiation (Figure 1A) and 410 nm in 15 min laser irradiation (Figure 1B), which is linked to the presence of specific chromophores or formation of radiation induced defects within materials. Additionally, a broad absorption band ranging from 400 to 500 nm was observed, which was not present in the UV–visible spectrum of the15 min laser irradiation (Figure 1B). This difference indicates the successful synthesis of laser irradiation.

#### 3.2.2 Fourier-transform infrared spectroscopy (FTIR)

**Figure 2:**
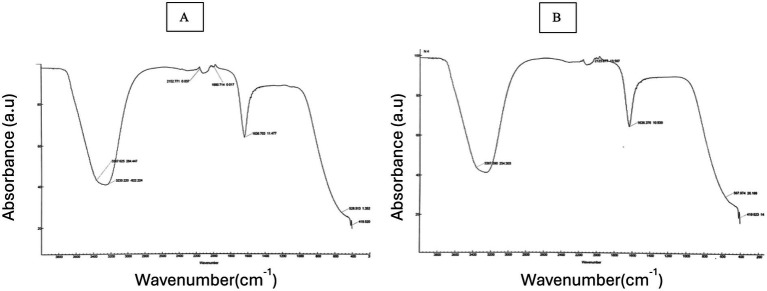
FTIR spectrum of **(A)** 10 min laser irradiation of *C. longa*, **(B)** 15 min laser irradiation of *C. longa.*

In order to verify the functional groups involved in the biosynthesis of laser irradiation, we used the FTIR spectrum as a qualitative instrument. Numerous functional groups, including as single, double, and fingerprint bonds, are seen in the Figure 2.

After 10 min of laser irradiation, the FTIR spectrum showed consistent and significant increase at 3368.625, 2152.771, 1990.714, 1636.703, 528.513, and 419.52 cm^−1^, as well as 3367.390, 2123.677, 1636.974, 567.974, and 419.623 cm^−1^. Polymeric hydroxyl compound O–H stretching, C–C stretching from phenyl groups, C=O stretching vibration, C–N stretching, enhancement of C–H and C–C vibrations, lipids, COO symmetric stretching, CH2 bending/stretching vibrations, C–O of mono-, 1112 oligo-, ester carbonyl (COOR), and carboxylate ion stretching (–COO–)–/C–O stretching vibration (amide), halo compounds, carbohydrates, and the pyranoid ring.

#### 3.2.3 Zeta potential determination

**Figure 3:**
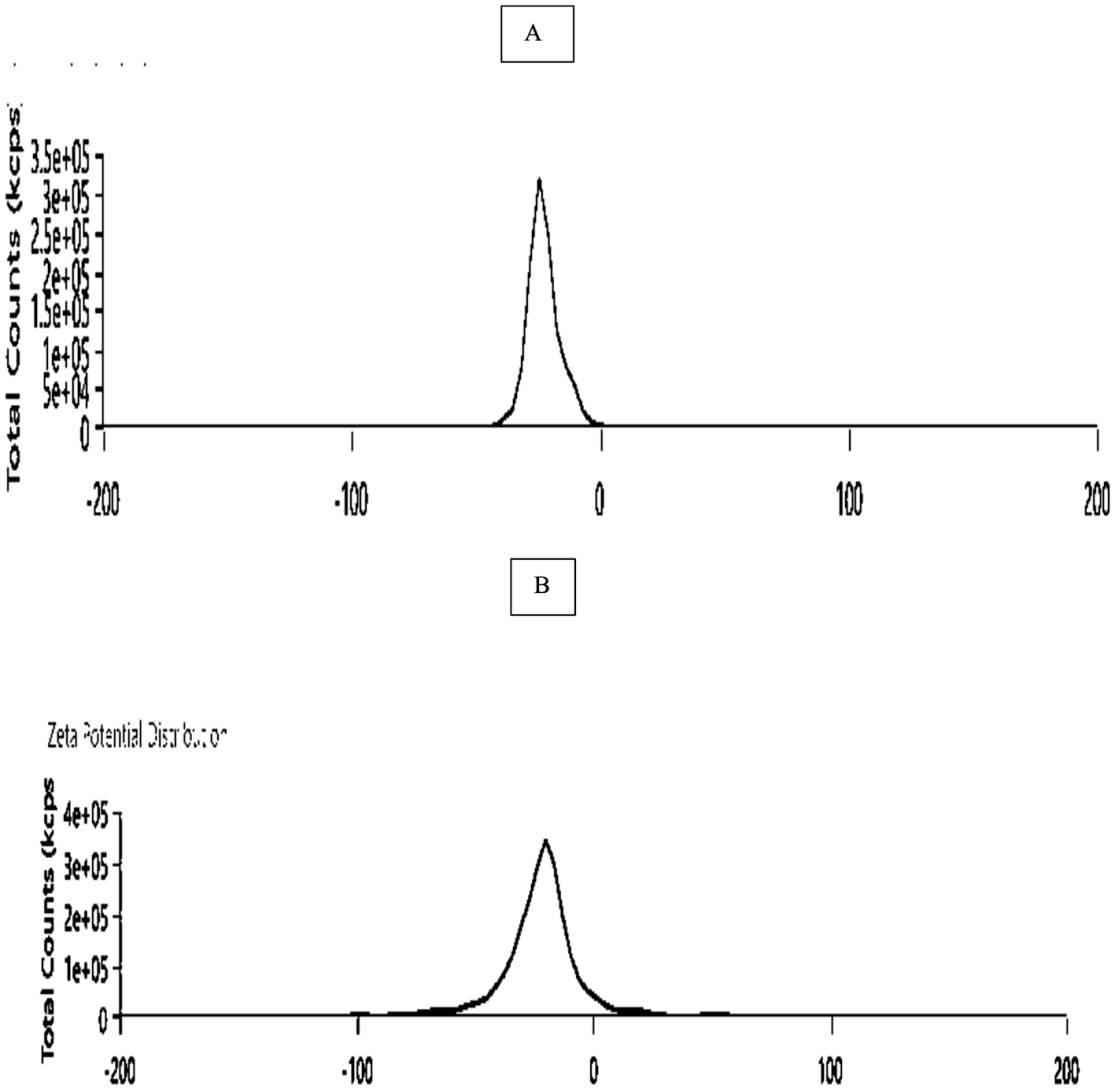
Zeta potential determination: **(A)** 10 min laser irradiation of *C. longa;* **(B)** 15 min laser irradiation of *C. longa.*

Zeta potential was used to assess the 10, 15 min laser irradiation of *C. longa*,’ surface charge (Figure 3). The bio-synthesized He–Ne laser-treated *Curcuma longa* were stable, as they had a value of−22.2 mV in 10 min laser irradiation and −25.4 mV in 15 min laser irradiation.

#### 3.2.4 Technical specifications of EDX

**Figure 4:**
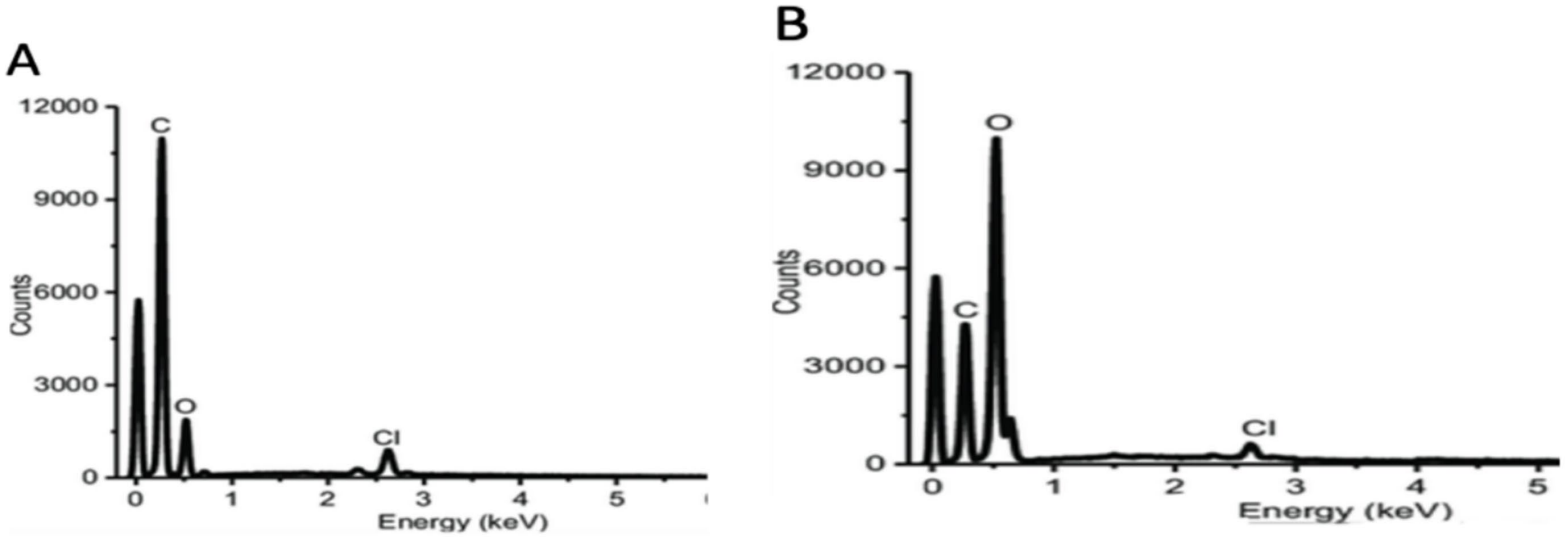
EDX spectra of **(A)** 10 min laser irradiation of *C. longa*, **(B)** 15 min laser irradiation of *C. longa.*

A high signal in the carbon and oxygen section of the EDX study showed that carbon and oxygen were biosynthesized (Figure 4). In both 10- and 15-min laser irradiation of *C. longa*, the proportion of carbon was 58%, followed by oxygen (33.5%) and chlorine (2.5%), which were present at a lower percentage and 49.6% in oxygen, respectively.

### 3.3 Impact of laser He–Ne laser treatments on the antimicrobial activity of Ethanolic curcumin extracts

**Figure 5:**
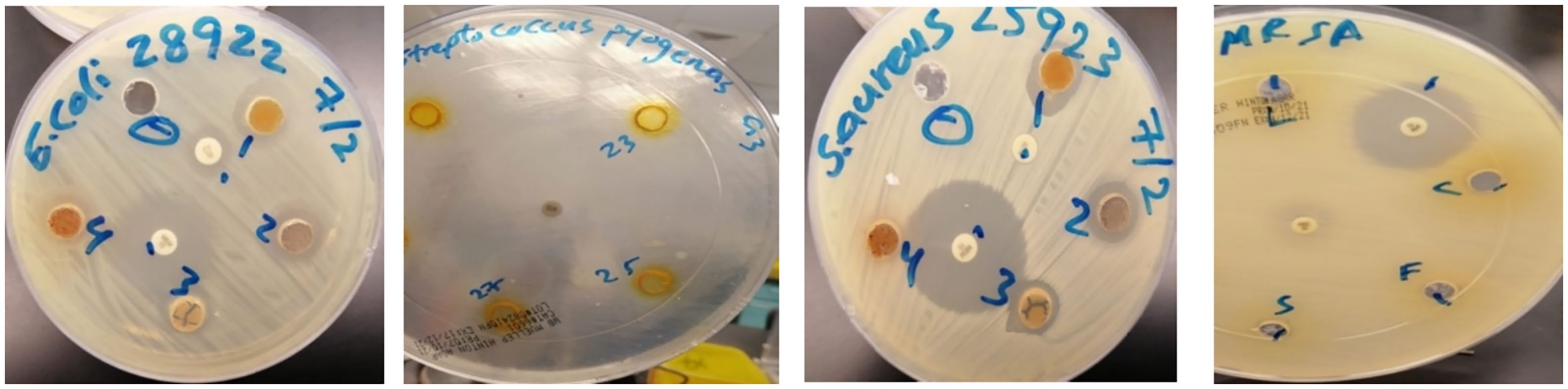
Inhibition zone diameters of curcuminoid extracts against tested bacterial strains. Data represent mean ± SD (*n* = 12). Statistical differences among treatments were analyzed using one-way ANOVA followed by Tukey’s HSD test. Different letters indicate significant differences at *p* ≤ 0.05.

*In vitro* disc diffusion assay was performed with ethanolic extracts of *C. longa* against the growth inhibition various bacterial strains (Table 2). The results revealed that He–Ne laser pretreatment of rhizomes for 10 or 15 min modulated the antibacterial efficacy of the extracts. Extracts from untreated control plants exhibited activity against all tested bacterial strains. Notably, extracts from rhizomes exposed to 15-min laser irradiation demonstrated enhanced antibacterial activity, particularly against *E. coli*, which corresponded with an increase in curcumin content. The observed activity against *E. coli* was nearly equivalent to that of 10 μg of imipenem, while activity against *S. aureus*, *MRSA*, and *S. pyogenes* remained similar to that of control extracts. In contrast, extracts from 10-min laser-treated rhizomes showed considerable reduction as anti-*E. coli* activity followed by controls but retained efficient antibacterial potential against other tested strains (Figure 5, Table 2). These findings indicate that the duration of He–Ne laser irradiation may enhance the antibacterial properties of curcumin extracts, with a pronounced effect against *E. coli*.

**Table 2:** Mean inhibition zone diameters (mm ± SD, *n* = 12).

Bacterial species	Laser treatment	Inhibition zone (mm)
*E. coli*	Laser 10 (a)	1.5 ± 0.3^b^
	Laser 15 (b)	2 ± 0.3^a^
	Control (curcumin)	1 ± 0.2^c^
	Imipenem, 10 μg/mL	1.5 ± 0.2^ab^
*S. aureus*	Laser 10	0.9 ± 0.2^c^
	Laser 15	1.3 ± 0.2^b^
	Control (curcumin)	0.8 ± 0.1^d^
	Imipenem, 10 μg/mL	1.5 ± 0.3^a^
*S. pyogenes*	Laser 10	1.2 ± 0.3^c^
	Laser 15	1.4 ± 0.3^b^
	Control (curcumin)	1 ± 0.2^d^
	Imipenem, 10 μg/ml	1.5 ± 0.2^a^
MRSA	Laser 10	1.3 ± 0.2^c^
	Laser 15	1.5 ± 0.2^a^
	Control (curcumin)	1.1 ± 0.1^d^
	Imipenem, 10 μg/ml	1.4 ± 0.3^b^

^*^Mean surface area of the inhibition zone (mm^2^) was calculated for each treatment after determining the mean diameter. This was calculated for all species in the same manner. Different letters within each row indicate significant differences (*p* ≤ 0.05, Fisher’s LSD test).

### 3.4 Evaluation of minimum inhibitory concentration and minimum bactericidal concentration of curcumin extracts

MIC and MBC values are presented in Table 3. Growth inhibition of *MRSA*, *E. coli*, and *S. pyogenes* occurred at 1 mg mL^−1^ across treatments. *S. aureus* displayed higher sensitivity with inhibition at 0.5 mg mL^−1^. Complete eradication (MBC) corresponded to MIC levels for all strains tested.

**Table 3:** MIC and MBC values (mg mL^−1^) of curcuminoid extracts against tested bacterial strains.

Treatment	*E. coli* MIC/MBC	*S. aureus* MIC/MBC	*S. pyogenes* MIC/MBC	*MRSA* MIC/MBC
Control	1/1	0.5/0.5	1/1	1/1
10 min laser	1/1	0.5/0.5	1/1	1/1
15 min laser	1/1	0.5/0.5	1/1	1/1

MIC and MBC values represent lowest concentration inhibiting or eliminating growth after 24 h.

### 3.5 Morphological changes by scanning Electron microscopy

Untreated *E. coli* cells maintained intact, smooth surfaces with regular rod morphology. Extracts obtained from laser-treated rhizomes induced visible membrane disruption. Cells exposed to 10-min extracts showed surface deformation, while 15-min treatment resulted in marked collapse and distortion of cell walls.

### 3.6 Antioxidant activity (DPPH assay)

Laser irradiation significantly enhanced radical scavenging capacity relative to untreated control (*p* ≤ 0.05). IC₅₀ values were lowest for 15-min extracts (0.69 mg mL^−1^), followed by 10-min extracts (0.71 mg mL^−1^). Values remained higher than gallic acid (0.15 mg mL^−1^).

### 3.7 Protein denaturation and anti-inflammatory activity

Laser-treated extracts exhibited significantly higher inhibition of protein denaturation than control at 500 μg mL^−1^ (p ≤ 0.05). Inhibition values reached 69 and 65% for 10- and 15-min treatments, respectively, compared with 55% for control.

### 3.8 Cytotoxicity assessment

Extracts showed dose-dependent reductions in MCF-7 cell viability at concentrations above 100 μg mL^−1^. No significant reduction in viability was observed at concentrations corresponding to MIC values.

## 4 Discussion

Post-harvest He–Ne laser irradiation significantly modified curcuminoid composition and enhanced functional properties of *C. longa* extracts. The most notable change was the increase in bisdemethoxycurcumin after 10–15 min exposure (Table 1), accompanied by partial recovery of demethoxycurcumin and stabilization of curcumin levels at 15 min. These selective changes align with prior work indicating that light-based elicitation can modulate secondary metabolite pathways in plant tissues (41–43). While underlying mechanisms were not directly evaluated, previous studies suggest that light-responsive metabolic regulation may involve photoreceptor signaling and downstream phenylpropanoid pathway activation (2, 44). The activation of the phenylpropanoid pathway is proposed here as a plausible mechanistic hypothesis rather than a confirmed pathway. Transcriptomic and enzymatic analyses will be required to validate this mechanism in *Curcuma longa*. Future work including transcriptomic or redox measurements will be required to confirm these pathways in *C. longa*.

**Figure 6:**
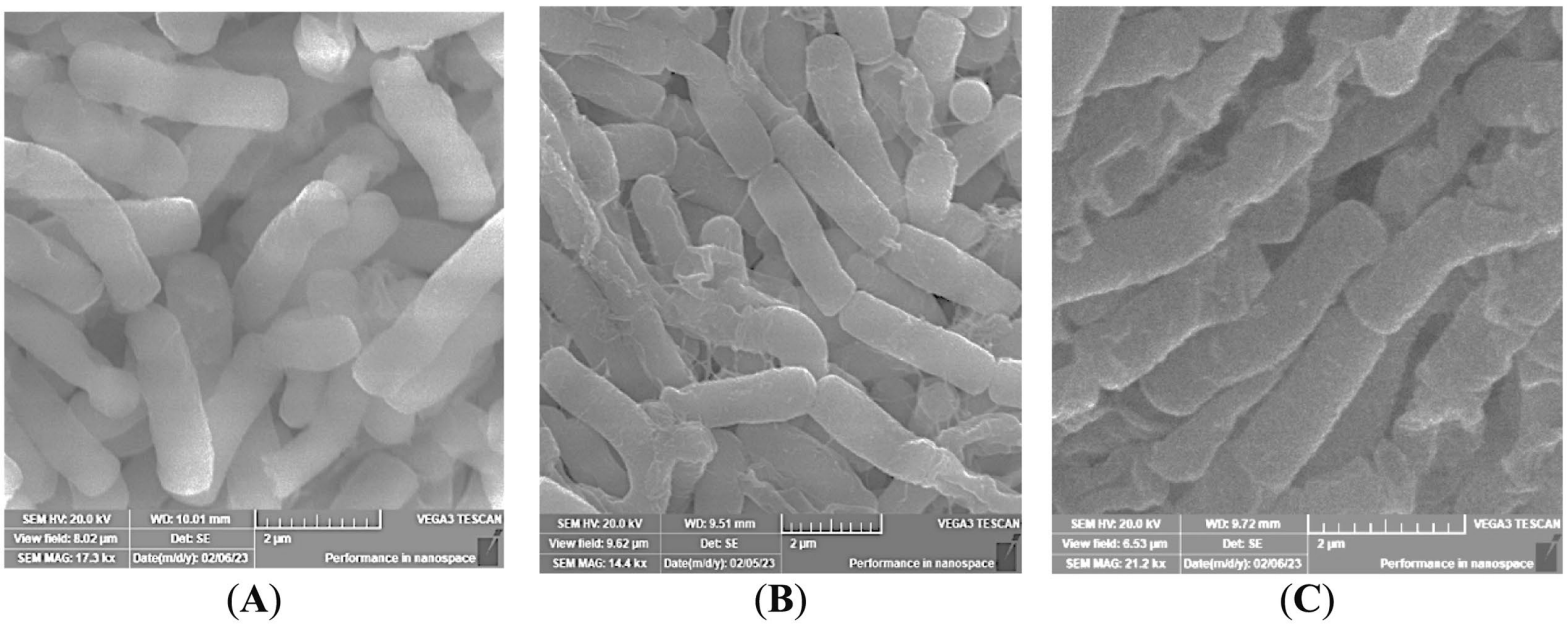
Representative SEM micrographs of *Escherichia coli* cells untreated (control) **(A)** or treated with curcuminoid extracts derived from 10- **(B)** and 15-min laser-irradiated rhizomes **(C)**. Images are representative of three independent experiments.

Laser irradiation also strengthened antibacterial activity, particularly against *E. coli*, with extracts from 15-min exposure producing the largest inhibition zones (Table 2, Figure 5). This enhancement parallels the higher curcumin and bisdemethoxycurcumin levels measured after 15 min (Table 1), consistent with reports linking curcuminoid enrichment to improved antimicrobial effects (45–47). MIC and MBC values remained similar across treatments for *MRSA*, *S. pyogenes*, and *E. coli* (Table 3), indicating that irradiation enhanced potency without altering bactericidal thresholds. SEM analysis confirmed visible membrane disruption in *E. coli* following exposure to extracts from laser-treated rhizomes (Figure 6), supporting the antimicrobial activity observed. These structural changes are consistent with established antibacterial mechanisms of curcuminoids, including membrane destabilization and oxidative stress–mediated damage (33, 48, 49).

**Figure 7:**
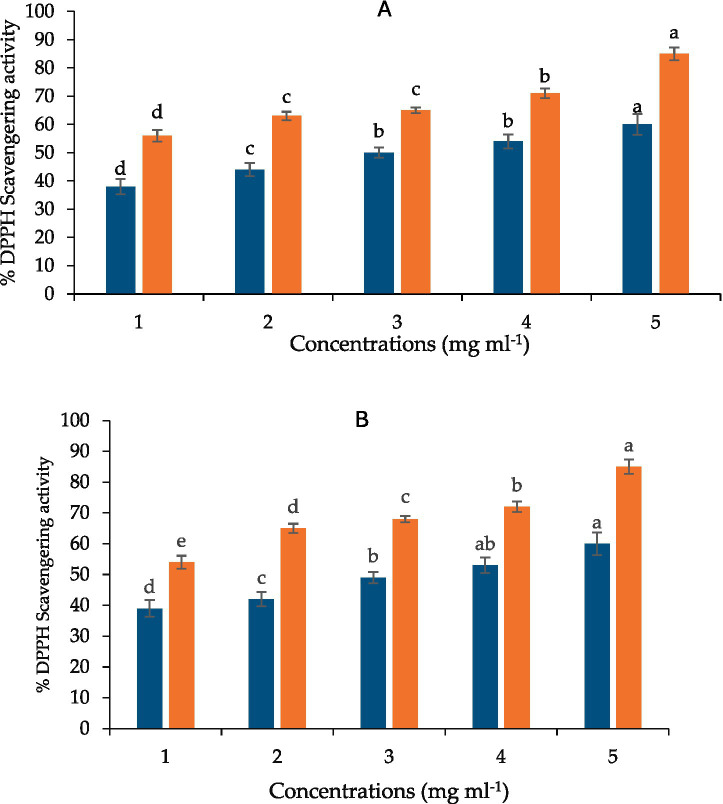
DPPH radical-scavenging activity of curcuminoid extracts from control, 10-min, and 15-min He–Ne laser-treated *Curcuma longa* rhizomes, compared with gallic acid, (orange is GA) and blue is extract **(A)** (10-min), and **(B)** (15-min) He–Ne laser-treated *Curcuma longa* rhizomes. Values are expressed as mean ± SD (*n* = 3 independent experiments). Statistical analysis was performed using one-way ANOVA followed by Tukey’s HSD post-hoc test. Different letters indicate statistically significant differences among treatments at *p* ≤ 0.05.

Laser-induced enrichment also increased antioxidant capacity, reflected by reduced IC50 values for DPPH scavenging at both irradiation durations (Figure 7). The IC50 values obtained (0.69–0.71 mg mL^−1^) fall within literature ranges for curcumin-rich extracts but represent an improvement over typical untreated material, which commonly shows IC50 values above 1 mg mL^−1^ (50, 51). These findings suggest that laser irradiation may enhance the value of turmeric as a natural antioxidant source for functional food applications (85).

**Figure 8:**
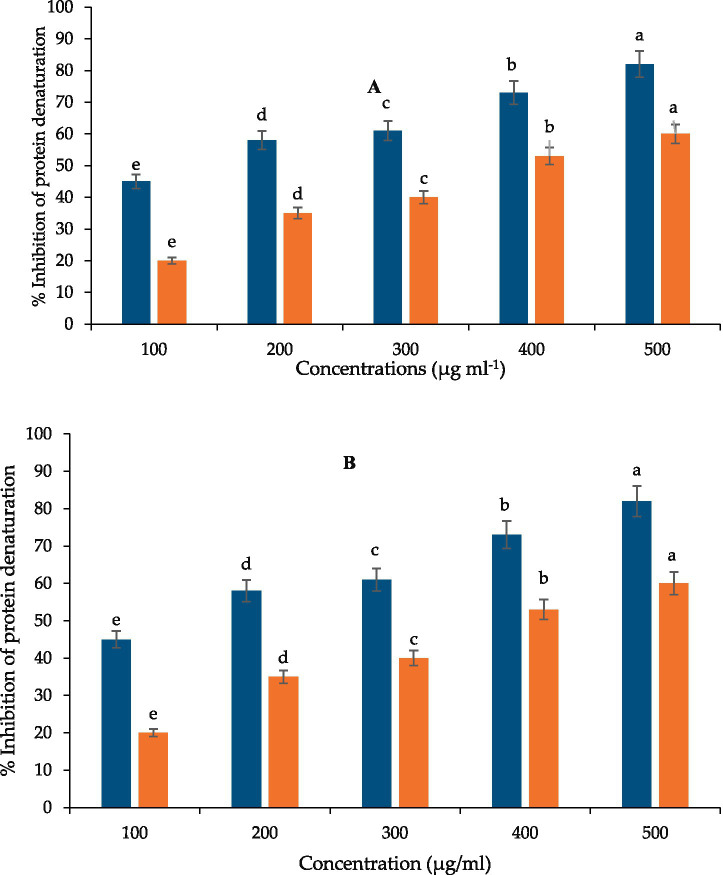
Inhibition of protein denaturation by curcuminoid extracts. Orange is *C. longa* extract and blue is *C. longa* **(A)** (10 min) and **(B)** (15 min) Ne–He *radiation*. Results are expressed as mean ± SD (*n* = 3). Statistical analysis was conducted using one-way ANOVA with Tukey’s HSD test, and different letters indicate significant differences at *p* ≤ 0.05.

**Figure 9:**
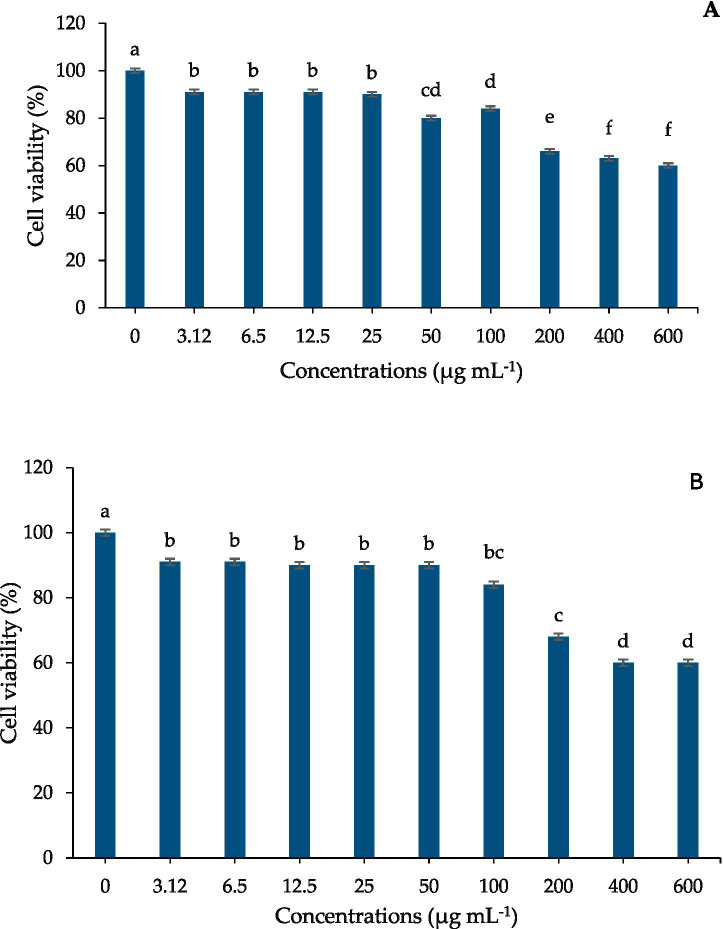
Viability of MCF-7 cells after exposure to curcuminoid extracts 10-min **(A)** and 15-min He–Ne laser-treated **(B)**. Data represent mean ± SD of three independent experiments (*n* = 3). Statistical differences relative to control were analyzed using one-way ANOVA followed by Tukey’s HSD test (*p* ≤ 0.05).

Anti-denaturation assays showed that extracts from laser-treated rhizomes produced higher inhibition of protein degradation than untreated extracts (Figure 8), indicating improved anti-inflammatory potential at non-cytotoxic concentrations. Importantly, cytotoxicity remained minimal at concentrations corresponding to MIC and antioxidant testing (Figure 9), suggesting potential suitability for food and nutraceutical applications that require low-toxicity bioactivity.

Overall, the combined biochemical and functional enhancements observed following He–Ne irradiation indicate that this non-thermal post-harvest method can improve extract composition and activity without compromising safety at relevant concentrations. These results extend previous reports on laser stimulation in medicinal plants by demonstrating post-harvest enhancement linked to functional outcomes rather than growth alone. Practical implications include the potential development of enriched turmeric extracts for natural preservation, antioxidant stabilization in food matrices, or value-added nutraceutical formulations (86). Future studies should evaluate extract performance in real-food systems and assess scalability of irradiation parameters for industrial integration.

The emergence of multidrug-resistant (MDR) bacterial strains presents a significant global health issue, undermining therapeutic efficacy and intensifying the pursuit of alternative antimicrobial strategies. MRSA is notably problematic among these pathogens because it can form protective biofilms, impede antibiotic penetration, and evade host immune responses (18, 52, 53). Conventional antimicrobials, although readily available, face significant limitations due to elevated production costs, environmental issues, and the rapid development of resistance mechanisms. This scenario highlights the necessity for innovative, sustainable, and low-toxicity antimicrobial solutions. Plant-derived bioactive compounds, particularly from medicinal and food-grade species, have garnered increasing attention due to their multifunctional properties, low toxicity, and potential applications in functional foods, nutraceuticals, and natural preservation systems (54–56). *Curcuma longa* is acknowledged for its antibacterial and antioxidant properties, primarily due to curcuminoids and related secondary metabolites (57–59). Increasing the levels or activity of these bioactives through sustainable technologies can facilitate the production of high-value functional ingredients appropriate for clean-label foods and environmentally friendly preservation systems. The impact of laser bio-stimulation on plant growth was initially recorded in 1970 (41), with subsequent studies across various plant species, including crops, vegetables, and herbs, corroborating these findings (40).

Compared to the control group, both the 10- and 15-min laser treatment groups exhibited a significant increase in plant height, root quantity, dried weight of leaves, roots, and rhizomes, as well as average rhizome diameters. The findings indicate that He–Ne laser pretreatment of rhizomes enhances the growth and yield of curcuma plants. Photosynthetic pigments in plants, such as chlorophyll a and b, exhibit a significant increase in correlation with plant development and rhizome production (60). Comparative analysis of irradiation treatments revealed that extracts obtained after 15 min of irradiation exhibited a significant inhibitory effect against *Escherichia coli*, while 10 min of irradiation resulted in greater inhibition of *Staphylococcus aureus* and *S. pyogenes*. Our research demonstrated a significant increase in antibacterial assays in both the 10- and 15-min laser treatment groups compared to the control group.

Laser irradiation has been identified as an effective method for modulating plant secondary metabolism, enhancing phytochemical concentrations, and improving extract functionality in the absence of chemical elicitors (61–63). This study shows that post-harvest He–Ne laser exposure effectively enhances the biochemical composition and functional activity of *C. longa* extracts in a sustainable manner.

The absorption spectrum of the synthesized laser irradiation exhibited increase at 335 nm after 10 min and 410 nm after 15 min of laser exposure, indicating the presence of specific chromophores or the formation of radiation-induced defects within the materials. The literature indicates that the excitation of chromophores or the formation vibrations of carbon or oxygen occurs within the range of 300–450 nm (64, 65). The results reveal a higher absorbance at 335 nm in 10 min laser irradiation (Figure 1A) and 410 nm in 15 min laser irradiation (Figure 1B), which is linked to the presence of specific chromophores or formation of radiation induced defects within materials. The FTIR spectrum indicated that the band at 3368.625 cm^−1^ for 10 min of laser irradiation and 3367.390 cm^−1^ for 15 min of laser irradiation corresponds to the vibrations of the OH group in phenolic compounds (Figure 2). The EDX analysis of the laser-irradiated sample revealed characteristic elemental increase between 4 and 5 keV, thereby confirming the presence of the corresponding metallic element in the synthesized nanostructures. The EDX spectrum exhibited a prominent elemental signal around 2 keV, thereby confirming the elemental composition (Figure 4). The negative zeta potential value signifies favorable colloidal stability of the nanoparticles, attributable to the capping and stabilizing phytochemical constituents inherent in the *C. longa* extract (Figure 3).

The antibacterial findings of this study indicate that laser-treated turmeric extracts exhibit enhanced inhibitory activity against *E. coli*, *S. aureus*, and *MRSA* in comparison to untreated controls. The 15-min laser treatment exhibited the most pronounced antibacterial effect, particularly against *E. coli*, which demonstrated larger inhibition zones compared to those seen with *S. pyogenes*. This pattern corresponds with earlier research indicating that curcumin-rich extracts generally show greater effectiveness against Gram-negative bacteria, attributed to improved membrane permeability and increased susceptibility to oxidative stress (66–70, 87). The improvement in antibacterial activity corresponds with the laser-induced elevation in curcumin and bisdemethoxycurcumin levels noted in the HPLC analysis (Table 1). These compounds demonstrate antimicrobial effects by disrupting membranes, interfering with protein and DNA synthesis, inhibiting quorum sensing, suppressing biofilm formation, and generating reactive oxygen species (71). Studies have reported analogous correlations where laser treatments improved the antimicrobial efficacy of extracts from various medicinal plants (72–74). This study did not directly measure ROS levels; however, the selective alterations in curcuminoid concentrations imply that photochemical pathways involving transient ROS formation may play a role in the modulation of curcuminoid biosynthesis and degradation during laser exposure. Prior research indicates that curcumin and its analogues can experience oxidative transformations facilitated by singlet oxygen, superoxide, and hydroxyl radicals under light irradiation (75). ROS involvement is suggested as a viable biochemical mechanism that aligns with the observed fluctuations, although direct verification is a goal for future research. The fluctuation in curcuminoid levels after laser treatment can be attributed to the opposing influences of irradiation-enhanced biosynthesis and photo-induced degradation. The mortality observed following 5-min laser irradiation may reflect an imbalance between laser-induced reactive oxygen species (ROS) generation and antioxidant defense activation. Short exposure may generate oxidative stress levels sufficient to damage cellular structures, whereas longer irradiation (10–15 min) may allow acclimation responses, including activation of antioxidant and secondary metabolic pathways. Similar laser-induced stress responses at short exposure durations have been reported in other plant systems (42).

The increase in curcuminoids and volatile constituents observed after 15 min of irradiation aligns with research indicating that light-based treatments, such as laser and LED exposure, promote the biosynthesis of secondary metabolites by activating photoreceptors and essential metabolic pathways (76–78). Studies on *Glycine max*, *Ocimum basilicum*, fennel, coriander, anise, and cumin indicate that laser irradiation enhances essential oil content, phenolic compounds, and antioxidant capacity (79, 80).

The red-light wavelengths emitted by the He–Ne laser may activate phytochrome-mediated signaling pathways. Red light is recognized for its role in regulating the synthesis of plant secondary metabolites through the activation of photoreceptors and subsequent modulation of genes in the phenylpropanoid pathway (81). Transcriptomic studies indicate that exposure to red LED light enhances the biosynthesis of phenolic and flavonoid compounds by upregulating phenylalanine ammonia-lyase (PAL) and 4-coumarate-CoA ligase (82). Laser irradiation markedly improved the antioxidant activity of *C. longa* extracts, evidenced by decreased IC50 values in the DPPH assay (0.71 and 0.69 mg mL^−1^ for 10- and 15-min treatments). The findings align with research indicating enhanced antioxidant potential subsequent to laser elicitation (83, 84).

He–Ne laser treatment represents a non-thermal, chemical-free, and low-energy method, positioning it as an environmentally sustainable technique for the post-harvest enhancement of plant materials. This study demonstrates that He–Ne laser treatment is an effective and sustainable elicitation strategy that significantly enhances the antimicrobial and antioxidant properties of *C. longa* extracts.

## 5 Conclusion

This research illustrates that post-harvest He–Ne laser irradiation is an efficacious, non-thermal method to improve the functional attributes of *C. longa* rhizomes. Laser irradiation for 10–15 min preferentially enhanced curcuminoids—especially bisdemethoxycurcumin—and augmented antioxidant capacity, antimicrobial activity, and anti-denaturation potential while preserving low cytotoxicity at useful concentrations. These modifications resulted in quantifiable increases in bioactivity without modifying MIC/MBC thresholds or jeopardizing safety, demonstrating that laser-treated extracts maintain their biological significance at suitable application levels.

The observed enhancements endorse the prospective use of He–Ne irradiation into processing methods designed for the production of natural antibacterial and antioxidant additives for clean-label food preservation, nutraceutical formulations, and active packaging systems. Laser irradiation, as a scalable, solvent-free, and residue-free technology, corresponds with sustainable manufacturing objectives and presents a feasible alternative to chemical elicitation or thermal enhancement techniques.

Subsequent research should verify the efficacy of enhanced extracts in actual food matrices, evaluate their durability during storage and processing, and refine irradiation conditions for industrial application. These initiatives will facilitate the conversion of laser-enhanced turmeric extracts into value-added, functional ingredients applicable in the food, health, and bioproduct industries. Direct measurement of ROS will be included in future work to confirm the proposed mechanism.

## Data Availability

The original contributions presented in the study are included in the article/supplementary material, further inquiries can be directed to the corresponding author.
